# A novel framework for noninvasive analysis of short-term atrial activity dynamics during persistent atrial fibrillation

**DOI:** 10.1007/s11517-020-02190-0

**Published:** 2020-06-13

**Authors:** Pietro Bonizzi, Olivier Meste, Stef Zeemering, Joël Karel, Theo Lankveld, Harry Crijns, Ulrich Schotten, Ralf Peeters

**Affiliations:** 1 Department of Data Science and Knowledge Engineering, P.O. Box 616, 6200 MD Maastricht, The Netherlands; 2 Université Côte d’Azur, CNRS, I3S, 2000, route des lucioles, Les Algorithmes - bât. Euclide B, 06900 Sophia Antipolis, France; 3 Department of Physiology, Maastricht University Medical Centre, P.O. Box 616, 6200 MD Maastricht, The Netherlands; 4 CARIM School for Cardiovascular Diseases, Maastricht University Medical Centre, P.O. Box 616, 6200 MD Maastricht, The Netherlands

**Keywords:** Electrocardiography, Atrial fibrillation progression, Atrial fibrillation substrate complexity, Propagation patterns, Recurrence analysis

## Abstract

ECG-based representation of atrial fibrillation (AF) progression is currently limited. We propose a novel framework for a more sensitive noninvasive characterization of the AF substrate during persistent AF. An atrial activity (AA) recurrence signal is computed from body surface potential map (BSPM) recordings, and a set of characteristic indices is derived from it which captures the short- and long-term recurrent behaviour in the AA patterns. A novel measure of short- and long-term spatial variability of AA propagation is introduced, to provide an interpretation of the above indices, and to test the hypothesis that the variability in the oscillatory content of AA is due mainly to a spatially uncoordinated propagation of the AF waveforms. A simple model of atrial signal dynamics is proposed to confirm this hypothesis, and to investigate a possible influence of the AF substrate on the short-term recurrent behaviour of AA propagation. Results confirm the hypothesis, with the model also revealing the above influence. Once the characteristic indices are normalized to remove this influence, they show to be significantly associated with AF recurrence 4 to 6 weeks after electrical cardioversion. Therefore, the proposed framework improves noninvasive AF substrate characterization in patients with a very similar substrate.

**Graphical Abstract:**
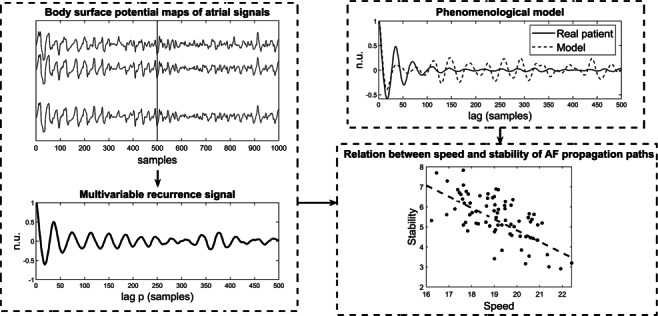
Schematic representation of the proposed framework for the noninvasive characterization of short-term atrial signal dynamics during persistent AF. The proposed framework shows that the faster the AA is propagating, the more stable its propagation paths are in the short-term (larger values of Speed in the bottom right plot should be interpreted as lower speed of propagation of the corresponding AA propagation patters).

Schematic representation of the proposed framework for the noninvasive characterization of short-term atrial signal dynamics during persistent AF. The proposed framework shows that the faster the AA is propagating, the more stable its propagation paths are in the short-term (larger values of Speed in the bottom right plot should be interpreted as lower speed of propagation of the corresponding AA propagation patters).

## Introduction

Quantification of the degree of electrophysiological remodelling in the atria during atrial fibrillation (AF), also addressed as AF substrate complexity, may form the basis for a more adequate AF stratification (not simply based on episode duration) and may provide useful information for guiding AF therapy [12, 13]. This remodelling influences the propagation of atrial activity (AA) wave fronts, suggesting that AF substrate complexity can be quantified from the (dis)organization of AA wave front propagation patterns (e.g. number of waves, atrial fibrillation cycle length (AFCL), conduction velocity). On the body surface, atrial signal dynamics during AF are reflected in the f-waves in the electrocardiogram (ECG), and previous studies have shown that parameters quantifying f-wave organization can be used to noninvasively determine AF substrate complexity [7, 21, 25]. This suggests that the ECG can be used to estimate AF progression and potentially guide therapy in clinical daily practice. However, the clinical use of the ECG for AF is still limited to its diagnosis. A possible reason is that ECG-derived AF substrate complexity does not represent sufficiently well the continuous spectrum of AF progression, and overlooks the subtle differences in very similar AF substrates (with similar degree of remodelling). However, patients with very similar AF substrate may still respond differently to the same treatment (for instance, to catheter ablation [11]).

In a previous study, we showed that propagation of AA during AF is a process characterized by different short- and long-term recurrent behaviours [18]. Short-recurrent behaviours are those related to a single cycle of propagation of an AA wave (or a single period, and thus related to the AFCL), while long-recurrent behaviours are those related to several cycles of propagation. A detailed description of those AA dynamics may help to better characterize the AF substrate heterogeneity and complexity. This requires understanding the origin of this recurrent behaviour, and linking it to the underlying mechanisms of AF. A starting point to unravel this link is to notice that since recurrence in time series data is closely related to repeatability of (oscillatory) components, recurrence is naturally linked to the frequency content of a signal. In another recent study, we speculated that the recurrent behaviour of AA signals, as recorded on the surface of the body, could be used to investigate the origin of the time-varying spatio-temporal properties of AA propagation during AF [6].

In this study, we formalize this approach and propose a framework for noninvasive characterization of atrial signal dynamics during persistent AF. For the sake of accuracy, this framework is developed by using high spatial resolution body surface potential map (BSPM) recordings. It proposes the construction of a signal which captures the recurrent behaviour of AA dynamics as reflected on the body surface, and it is able to separate the short- and long-term recurrent behaviour in the AA oscillatory patterns. The characteristic indices which can be extracted from such signals are then correlated to the spatial variability of body surface AA signals. This allows to speculate on the link between those indices and the underlying degree of electro-structural remodelling, and test the hypothesis that the variability in the oscillatory content of AA is due mainly to a spatially uncoordinated propagation of the AF waveforms. A simple phenomenological (not mechanistic) model of AA dynamics is proposed to investigate the validity of this hypothesis, and to reveal an influence of the AF substrate complexity on the short-term recurrent behaviour of AA propagation. Removal of this influence from the aforementioned characteristic indices helps strengthen their association with AF recurrence 4 to 6 weeks after electrical cardioversion (after adjusting for state-of-the-art AF complexity parameters [7, 25]), and supports the idea that the proposed framework allows for a detailed noninvasive analysis of AA propagation dynamics during AF.

## Materials and methods

### BSPM-dataset

In this study, we employed a dataset of body surface potential maps (BSPM) recorded in 75 patients in persistent AF (retrieved from Maastricht University Medical Centre, Maastricht, the Netherlands; the study was approved by the institutional ethics review board). BSPM were recorded with 120 anterior and 64 posterior leads (ActiveTwo BSM Panels Carbon Electrodes, Biosemi B.V., The Netherlands). All patients underwent electrical cardioversion, and 32 out of 63 patients with follow-up showed AF recurrence 4 to 6 weeks after procedure. ECGs were sampled at 2048 Hz, and downsampled to 256 Hz. A 1-min segment was selected for each subject, low-quality leads were excluded (low signal-to-noise ratio, poor electrode contact, motion artefacts), and Wilson’s Central Terminal was subtracted in line with conventional ECG analysis. All signals were band-pass filtered between 1 and 100 Hz (3rd-order Chebyshev), and QRST cancellation was performed using an adaptive singular value decomposition method, inspired by the approach in [1], with multiple QRST window templates defined using hierarchical clustering. The extracted AA signals were post-filtered with a zero-phase notch filter at 50 Hz (2nd-order IIR filter) to suppress power line noise, and with a 3 Hz zero-phase high-pass filter (3rd-order Chebyshev) to remove low-frequency residuals not related to (persistent) AF.

### Multi-variable AA recurrence signal

**Fig. 1:**
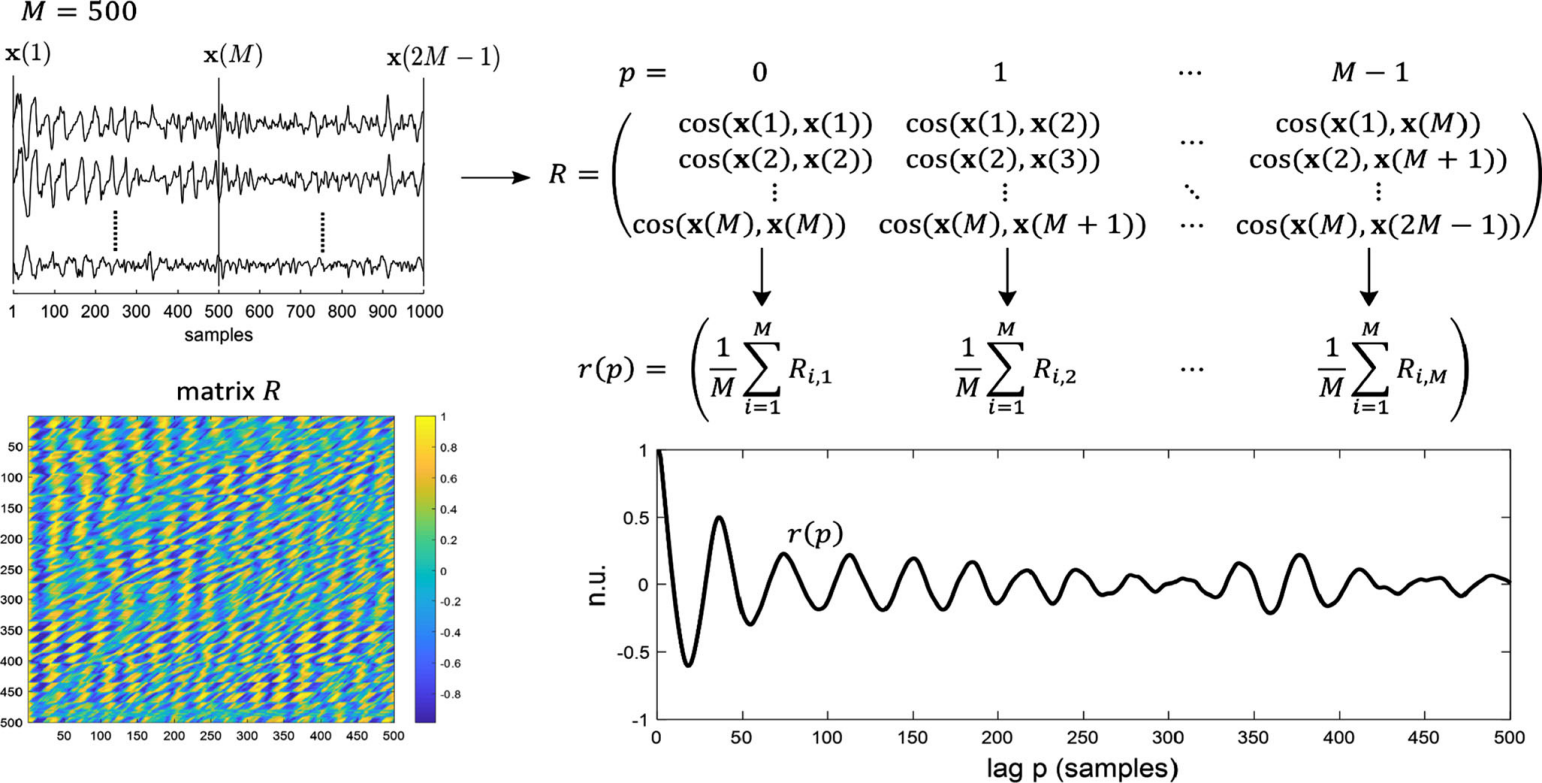
Schematic representation of the construction of a multi-variable AA recurrence signal *r*(*p*), starting from AA signals extracted from ECG recordings. A visualization of the matrix *R* is also provided (bottom-left)

In [18], we introduced an approach to investigate the nonstationary properties of noninvasive AA signals based on their recurrence behaviour. Given a matrix *X* of size *ℓ* × *N* collecting all extracted AA signals from a patient (*ℓ* leads, and *N* samples), each column of *X* is assumed to provide an *ℓ* × 1 vector **x**(*n*), *n* = 1, … , *N*, which represents the overall spatial AA from all electrodes (hence *multi-variable*) at a given time instant. Then, a square matrix *R* of size *M* × *M* is generated by computing:

1 $$ R_{i,j} = \frac{{\mathbf x}(i)^{T}{\mathbf x}(i+j-1)}{||{\mathbf x}(i)||_{2} ||{\mathbf x}(i+j-1)||_{2}}, \parbox{15em}{with $ i = 1,\ldots,M$, \\{with}$ j = 1,\ldots,M$} $$

where *M* is the window size of the analysis (*M* ≤ *N*), chosen to capture self-similar behaviour in AA propagation. Each entry of *R* is therefore a measure of the cosine of the angle between two vectors ($R_{i,j}=\cos \limits ({\mathbf x}(i),{\mathbf x}(i+j-1))$), and provides a correlation-based distance which focuses on the shapes of the signal profiles, rather than their magnitudes. This allows to obtain small angles (high similarity) for spatial wave fronts with similar morphologies even when their corresponding amplitudes are different, in order to highlight the presence of self-similar patterns in the data. Moreover, column *j* includes correlation values at lag *p* = *j* − 1. The average over each column (per lag) provides a multi-variable autocorrelation function of the spatial AA oscillatory patterns, for lags *p* = 0, … , *M* − 1, which is defined as the multi-variable AA recurrence signal *r*(*p*) for a patient. This signal allows to investigate the temporal recurrence of global AF spatial patterns over the all body surface covered by the BSPM electrodes. Figure 1 provides a schematic summary of the procedure used to construct the signal *r*(*p*). Recurrence is expected to be high when similarity between two vectors **x**(*i*) and **x**(*i* + *j* − 1) is high (or equivalently, *R*_*i*,*j*_ is close to one). For instance, under the hypothesis that a hypothetical AF cardiac dipole passes through the same points after a period *T* (with *T* expressed in samples), the cosine of the angle between any two vectors **x**(*i*) and **x**(*i* + *T*) which are *T* samples apart is expected to equal 1. Equivalently, a value of − 1 for the cosine of the angle is expected to be reached when the two vectors are in opposition of phase, or half a period (i.e. $\frac {T}{2}$ samples) apart. Therefore, a multi-variable AA recurrence signal captures the spatial similarities of AA propagation patterns (in terms of spatial correlation) over time, and it exploits the spatial information in terms of spatial similarity/diversity.

**Fig. 2:**
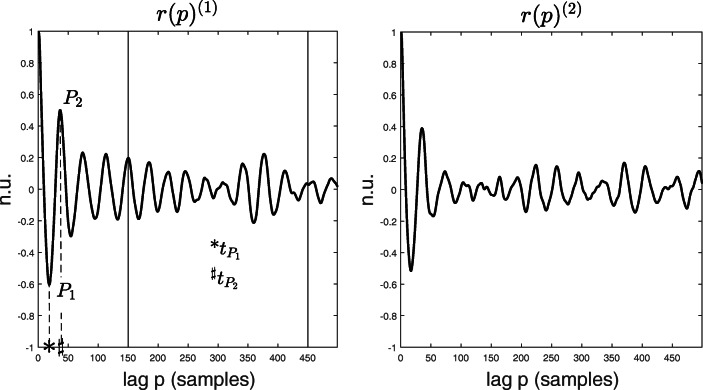
*r*(*p*)^(*m*)^ curves from two consecutive blocks in a patient (n.u. is normalized units). The characteristic indices *P*_1_, *P*_2_, $t_{P_{1}}$, and $t_{P_{2}}$ are shown on the left plot, with the two vertical lines defining the interval for the computation of the LTR index

This procedure is repeated over nonoverlapping subsets of columns of *X*, called blocks, each block *m* including 2*M* columns. A matrix *R*^(*m*)^ and the corresponding multi-variable AA recurrence signal *r*(*p*)^(*m*)^ can then be computed for each block *m*, to account for nonstationary recurrence behaviours in AF propagation (*p* = 0, … , *M* − 1; $m=1,\ldots ,\left \lfloor \frac {N}{2M}\right \rfloor $). The reason for looking at consecutive nonoverlapping blocks is that we consider it sensible to assume that a self-similar behaviour in AA propagation is likely not to last over a long time interval, and we expect breaks in the phase of the AF patterns to occur (piecewise stationarity). We assume the number of those breaks to be positively correlated with AF progression and electro-structural remodelling of the myocardium. It is difficult to know in advance what could be a suitable window size *M* for a patient. The smaller *M* is the better nonstationarities can be handled, but the more dissimilar *r*(*p*)^(*m*)^ will be over consecutive blocks, and vice versa. A value of *M* = 500 was used in this study, which guarantees to span approximately 4 s (i.e. 2*M* columns) in each block (at a sampling frequency of 256 Hz), thus capturing several periods of AF [18]. An example of multi-variable AA recurrence signals *r*(*p*)^(*m*)^, computed over two consecutive blocks, is given in Fig. 2. Notice the variability in the shape of *r*(*p*)^(*m*)^ from block to block (especially for larger lags), underlying the nonstationary behaviour of AF propagation patterns. AF maintenance has been explained by different conceptual models: repeated rapid focal activity [15], rotors [10], or disrupted conduction of multiple stable wavelets that become fragmented [3, 4]. These mechanisms are charaterized by different degrees of periodicity and organization (with rapid focal activity and rotors expected to produce more periodic and organized wave front propagation patterns than multiple wavelets), and therefore are expected to be featured differently on multi-variable AA recurrence signals *r*(*p*)^(*m*)^ (with the first two showing stronger long-term recurrence than the latter). This suggests that the proposed framework should be able to handle different mechanisms of maintenance of AF. Three characteristic indices associated with an *r*(*p*)^(*m*)^ signal are also introduced:

- Long-term recurrence (LTR): computed as the average over all blocks of the mean absolute value of the portion of an *r*(*p*)^(*m*)^ signal comprised between 150 ≤ *p*≤ 450 (portion within the two vertical bars in Fig. 2 left). In [18] we showed that the envelopes of *r*(*p*)^(*m*)^ signals are characterized in general by two distinct behaviours: an early phase characterized by a decreasing autocorrelation value (for small values of the time lag *p*), and a later phase characterized by an approximately constant behaviour, for values of *p* in the range 150 ≤ *p*≤ 450 (Fig. 3 in [18]). This suggests that the propagation of AA during AF is a process characterized by different short- and long-term recurrent behaviours. LTR is therefore considered a measure of long-term recurrent behaviour of AA propagation in a patient, and it is assumed to be related to the overall AF substrate complexity (with a higher LTR value to be related to a lower degree of electro-structural remodelling).
- The absolute value of the first negative peak |*P*_1_| and the first positive peak *P*_2_ in an *r*(*p*)^(*m*)^ signal (Fig. 2 left): they can be interpreted as a measure of the strength of short-term recurrent behaviour of AA propagation in a patient (with higher values of |*P*_1_| and *P*_2_ being related to a lower degree of electro-structural remodelling). *P*_1_ is expected to occur at approximately half a period of the AA propagation waveform ($\sim $ half of the AFCL), while *P*_2_ at approximately a full period ($\sim $ one AFCL).
- $t_{P_{1}}$ and $t_{P_{2}}$: time lags corresponding to *P*_1_ and *P*_2_, respectively. They are expected to be correlated with the AF dominant frequency (or the AFCL, with higher values of $t_{P_{1}}$ and $t_{P_{2}}$ being related to a lower AF dominant frequency, and thus a lower degree of electro-structural remodelling).

### Spatial variability of AA propagation

**Fig. 3:**
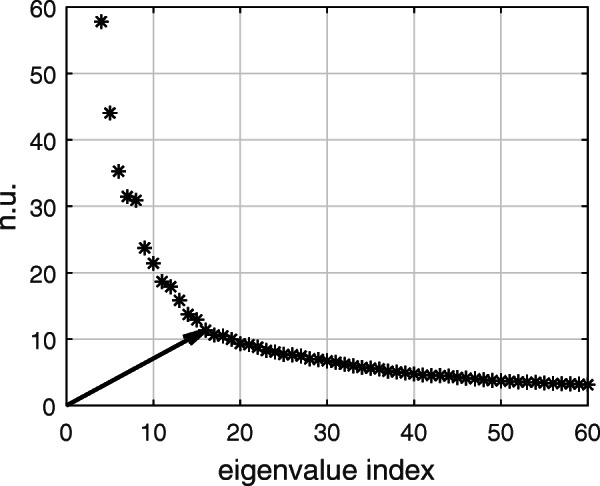
Example of calculation of SVAAP from the spectrum of a matrix. Indicated with an arrow is the point that minimizes the distance with the origin. n.u., normalized units (by scaling all singular values by $\frac {\ell }{\sigma _{1}}$)

Given that a multi-variable AA recurrence signal *r*(*p*)^(*m*)^ contains information about the temporal repetitiveness of spatial AA oscillatory patterns on the body surface, a quantification of the organization of those patterns could be used to provide an interpretation of *r*(*p*)^(*m*)^ signals, and its characteristic indices. This can be achieved by looking at the amount of spatial variability in AA propagation (SVAAP), as reflected on surface AA signals. In [6] we proposed to measure SVAAP from the complexity of surface AA signals. SVAAP is assessed by computing the AA subspace dimension, which is defined starting from identifying the point in the spectrum of matrix *X* (i.e. the set of its eigenvalues) that minimizes the distance between the plot of the spectrum and the origin (in a similar way to how the best cutoff point on a receiver operating characteristic (ROC) curve is identified; see Fig. 3). Once this point is identified, its x-coordinate represents the index of the corresponding eigenvalue. Since this eigenvalue can be considered as the first negligible eigenvalue of *X* (being at the corner of the plot of the spectrum), the index of the previous eigenvalue is then chosen as a measure of SVAAP. Thus, if we assume that the vector **v** = [*x*,*y*]^*T*^ identifies a generic point in the plot of the spectrum of *X*, SVAAP can be defined as:

$$ \text{SVAAP} = \underset{x}{\text{argmin}} ||{\mathbf v}||_{2} -1. $$

The spectrum of *X* is generated by applying singular value decomposition to *X*, and then by scaling all singular values by $\frac {\ell }{\sigma _{1}}$ (where *σ*_1_ is the first singular value). Scaling ensures to have same units on both axes of the spectrum, and accurately estimate SVAAP. SVAAP is assessed over short (*s*-SVAAP) and long (*l*-SVAAP) AA segments. *l*-SVAAP is computed over nonoverlapping 5-s segments (i.e. focusing on portions of *X* of size (*ℓ* × *F*_*s*_ ⋅ 5), with *F*_*s*_ the sampling frequency), while *s*-SVAAP is computed over nonoverlapping segments of *q* samples (with $q=\frac {F_{s}}{f_{AF}}$, where *f*_*AF*_ is the AF dominant frequency, and focusing on portions of *X* of size (*ℓ* × *q*)), and then averaged over all segments. This choice for *q* allows to have an adaptive way to set the segment length in a patient, to cover a time span of about a full cycle of the overall AA propagation. Patients with persistent AF have been shown to be characterized by unstable patterns of activation (including wave fronts and disorganized activity) and by narrow fibrillation waves [3, 14]. Based on those observations, we assume that the variability in the oscillatory content of AA is due mainly to a spatially uncoordinated propagation of the AF waveforms, and we expect a patient affected by persistent AF to show *l*-SVAAP > *s*-SVAAP.

### Phenomenological model of atrial signal dynamics during persistent AF

**Fig. 4:**
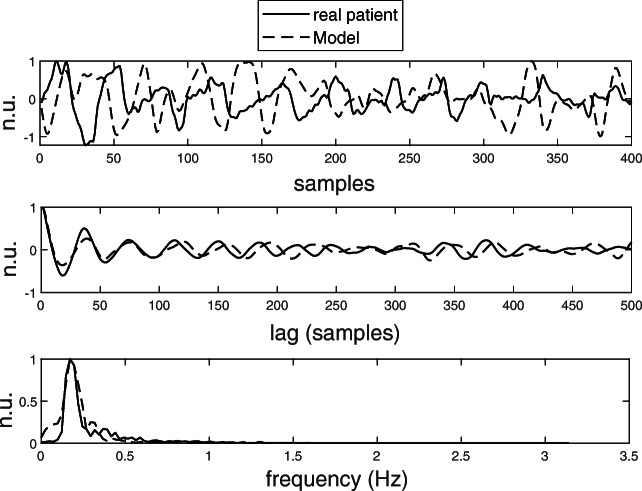
Examples of real and simulated pseudo-AA signal (top; only the first 400 samples are displayed), *r*(*p*)^(*m*)^ and $\hat {r}(p)^{(m)}$ signals (middle), and amplitude spectra (bottom), from a patient and from model (2), with *d* and *v* optimized on the patient as described in the main text

A simple phenomenological model of atrial signal dynamics as observed on the body surface is proposed to investigate the hypothesis introduced at the end of the previous section. The model is able to generate pseudo-AA signals that can replicate the overall behaviour of *r*(*p*)^(*m*)^ signals computed on patients’ data (Fig. 4 middle). The model assumes a process with time-varying spatial properties of AA propagation. In [6], we showed that this assumption reflects more accurately what is observed in real patients (*s*-SVAAP < *l*-SVAAP, see Section 3.1), than its counterpart (a process with stationary spatial properties of AA propagations and time-varying frequency properties only, for which *s*-SVAAP ≈ *l*-SVAAP is expected). The model is given in (2):

2 $$ \begin{array}{llll} m_{k}(n) = \cos(2\pi\frac{f_{AF}}{F_{s}}n+s_{k}^{[d,v]}(n)+k/2), \\ \text{with}\ s_{k}^{[d,v]}(n)\ \text{being a random walk process.} \end{array} $$

*F*_*s*_ is the sampling frequency, and *k* = 1, … , *ℓ* allows to generate AA propagation loops in a *ℓ*-D space. For instance, *ℓ* = 3 allows to generate 3-D loops which simulate the AA cardiac dipole during AF (also in case no randomness is introduced). $s_{k}^{[d,v]}(n)$ is a stochastic process where each point is randomly drawn from a normal distribution, such that:

3 $$ \begin{array}{llll} s_{k}^{[d,v]}(n) =s_{k}^{[d,v]}(n-1)+{\Delta}_{k}^{[d,v]}(n), \\ \text{with} {\Delta}_{k}^{[d,v]}(n) \sim N(0,v^{2}), \text{s.t.} \lvert s_{k}^{[d,v]}(n) \rvert \leq d. \end{array} $$

Hence, *d* controls the range of *s*(*n*) (the larger *d* the larger the range), while *v* controls the rate of variation of the increment (the larger *v* the larger the rate of variation). The use of a stochastic component in (2) is suggested by what can be observed on real AA signals during AF. Indeed, for the same set of parameters (*d*,*v*) (same AF substrate), model (2) will give different AA dynamics over different runs, as for the different dynamics of AF propagation captured by *r*(*p*)^(*m*)^ over consecutive blocks in the same patient (Fig. 2). It should be recalled that the instantaneous frequency of a pure cosine is the derivative with respect to time of its argument or phase. Then the model introduced in (2) exhibits also a time-varying frequency because of the stochastic process. If this stochastic process is the same regardless the value of *k* then, for *ℓ* = 3, the corresponding 3-D loops lie all on the same plane, whereas if it differs with *k*, the 3-D loops will span the space differently, in agreement with our hypothesis. In both cases, the instantaneous frequency will vary with time.

The *ℓ*-D *m*_*k*_(*n*) pseudo-AA signals in (2) are then linearly mapped into a 184-D space to simulate AA signals from BSPM recordings (mapping obtained by means of a 184 × *ℓ* random matrix). These simulated AA signals are in turn used to investigate the hypothesis. Figure 4 top and middle show the pseudo-AA signal and $\hat {r}(p)^{(m)}$ signal, obtained by fitting (2) to a patient from the BSPM-dataset (where the $\hat {}$ shows that this is assessed from the model; more details about how optimal values for the parameters *d* and *v* for a specific patient were obtained are provided in Section 2.4.1). Figure 4 bottom shows that the corresponding pseudo-AA signals in (2) give amplitude spectra similar to those we observed in persistent AF patients (with no strong harmonic components). It is important to remark that, when fit to a patient, the model is expected to give similar dynamics of their AA signals, and not to replicate exactly the morphologies of the AA signals and the *r*(*p*)^(*m*)^ signal, which explains the different morphologies in Fig. 4 top and middle.

The model is simple as it only generates a single sinusoidal waveform, with phase modulation. In this respect, it does not aim to be a mechanistic model that reproduces the electrophysiological substrate of AF characterized by multiple interacting waveforms wandering in the atria, nor it accounts directly for the heterogeneity of the atrial tissue. It mainly aims to be a descriptive model which allows to replicate the effects of the AF substrate on the body surface as observed on persistent AF patients (as shown from the model output and the corresponding $\hat {r}(p)^{(m)}$ signal in Fig. 4), to be able to draw conclusions on the global spatio-temporal behaviour of AF propagation patterns. As a consequence, the *s*-SVAAP and *l*-SVAAP values of the 184-D simulated AA cannot theoretically exceed *ℓ*, while the corresponding values computed on real signals can be greater than *ℓ* (with *ℓ* equal to the median *l*-SVAAP observed on patients).

#### Fitting the model to patients’ data

The following approach was used to find realistic values for parameters *d* and *v* in (2) which could generate pseudo-AA signals with $\hat {r}(p)^{(m)}$ signals similar to those observed in real patients. For each of the 75 patients from the BSPM-dataset, and given a specific pair of parameters *d* and *v*, 40 different models were generated, and linearly mapped to a 184-D space. From each model output, the average $\hat {r}(p)^{(m)}$ signal was obtained and the corresponding $|\hat {P}_{1}|$, $\hat {P}_{2}$, and $\hat {\text {LTR}}$ were computed, and compared with those from a patient by measuring the sum of the absolute differences. For each patient, the average AF dominant frequency computed over all leads was used as input for *f*_*AF*_ in (2). Additionally, for each pair of *d* and *v* parameters, *f*_*AF*_ was varied over the range [*f*_*AF*_ − 1,*f*_*AF*_ + 1] Hz, with step 0.5 Hz, to account for variability in the AF dominant frequency. *d* was varied over the range [6,14] (with step 1), and *v* over the range [0.16,0.32] (with step 0.02). All possible combinations of the two parameters were tested. The ranges were defined by empirically varying *d* and *v* and observing what ranges provided realistic AA dynamics. For each patient, the pair (*d*,*v*) which minimized the average sum of the absolute differences was selected. The resulting median(IQR) of range *d* and rate *v* were 8.0(3.8) and 0.30(0.06), respectively.

### Analyses

#### Correlation between spatial variability of AA propagation and AA recurrence

Correlation between spatial variability of AA propagation and AA recurrence was investigated on the BSPM-dataset. We also looked at the relation between *l*-SVAAP and *s*-SVAAP, to test the hypothesis that the variability in the oscillatory content of AA is due mainly to a spatially uncoordinated propagation of the AF waveforms (results are reported in Section 3.1)

#### Assessing SVAAP on the model’s output

*s*-SVAAP and *l*-SVAAP were then assessed on the pseudo-AA signals generated by means of (2), with *ℓ* = 15 (based on the median value of *l*-SVAAP obtained on patients; see Section 3.1)). For each of the 75 pairs of optimal parameters *d* and *v* identified for each patient, 100 different models were generated (and linearly mapped to a 184-D space). *s*-SVAAP and *l*-SVAAP were then assessed on the output of each model and averaged over the 100 simulations, to give an average value of *s*-SVAAP and *l*-SVAAP per *d* and *v* pair (result are reported in Section 3.2).

#### Relation of |*P*_1_| and *P*_2_ with LTR

The proposed phenomenological model was used to investigate the relation of |*P*_1_| and *P*_2_ with LTR, for a fixed value of *f*_*AF*_, and reveal (at a simulation level) a possible influence of the AF substrate complexity on the short-term recurrent behaviour of AA propagation. For this, *d* and *v* were varied again over the range [6,14] (with step 1) and [0.16,0.32] (with step 0.02), respectively. For each parameter combination, 20 different models were generated, and linearly mapped to a 184-D space. $\hat {P}_{1}$, $\hat {P}_{2}$, and $\hat {\text {LTR}}$ were computed from the corresponding $\hat {r}(p)$ curves, and averaged over the 20 simulations. This analysis was repeated for *f*_*AF*_ equal to 5, 6.5, and 8 Hz. These values were chosen based on the range of *f*_*AF*_ observed in the patients. After the influence of the AF substrate complexity on the short-term recurrent behaviour of AA propagation was revealed, |*P*_1_| and *P*_2_ were divided by LTR, to produce normalized indices $|\tilde {P}_{1}|$ and $\tilde {P}_{2}$. This was done to remove the influence of the AF substrate complexity on the short-term recurrent behaviour, and allow to better compare among patients by bringing them to a similar level of complexity, and in turn to better interpret the short-term recurrent behaviour of AA propagation during AF (results are reported in Section 3.3)

#### Relation of $|\tilde {P}_{1}|$ and $\tilde {P}_{2}$ with AF recurrence after electrical cardioversion

Finally, given that $|\tilde {P}_{1}|$ and $\tilde {P}_{2}$ are expected to carry information on short-term AA dynamics during AF, we investigated their relation with AF recurrence 4 to 6 weeks after electrical cardioversion, in order to infer a possible association. Assuming that patients showing AF recurrence after cardioversion are characterized by a more complex AF substrate (or equivalently, a more progressed electro-structural remodelling), we would expect this to be reflected in a lower $|\tilde {P}_{1}|$ and $\tilde {P}_{2}$ (less self-similar waveform morphologies on the short term). We also looked at other state-of-the-art ECG-based indices of AF complexity and investigated their association with AF recurrence 4 to 6 weeks after electrical cardioversion. Based on our previous work on standardization of noninvasive indices for the assessment of AF complexity [7, 22, 25], we selected the following parameters: spatial complexity, variability of spatial complexity, spatio-temporal stationarity, dominant frequency, spectral concentration and spectral variability, multivariate organization index, multivariate spectral entropy, sample entropy, f-wave amplitude, f-wave power, relative harmonic energy. These parameters differ in terms of domain of analysis (time-based vs. frequency-based), and use of temporal and/or spatial information. Some of these parameters are multi-lead based, while others are single-lead based. For the single-lead based parameters, parameters were computed on each lead, and the average over all leads was taken. We refer the interested reader to the studies cited in [7, 25], for a definition of these parameters, and their relation with AF substrate complexity and the degree of electro-structural remodelling in the atria. A univariate logistic regression model was generated for $|\tilde {P}_{1}|$ and $\tilde {P}_{2}$ and each state-of-the-art parameters to assess their association with AF recurrence after electrical cardioversion. Moreover, a multi-variable logistic regression model was also generated with all state-of-the-art parameters and $|\tilde {P}_{1}|$ and $\tilde {P}_{2}$ as input. Finally, the univariate analysis for $|\tilde {P}_{1}|$ and $\tilde {P}_{2}$ was repeated on the 12-lead ECG recordings extracted from the corresponding BSPM recordings, to investigate whether the information carried by the 12-lead ECG is still sufficient to accurately determine $|\tilde {P}_{1}|$ and $\tilde {P}_{2}$ and provide a possible association with AF recurrence after electrical cardioversion (results are reported in Section 3.4). This is relevant as the 12-lead ECG is considered the standard tool to assess the electrical functioning of the heart in the clinics.

## Results

### Correlation between spatial variability of AA propagation and AA recurrence

**Fig. 5:**
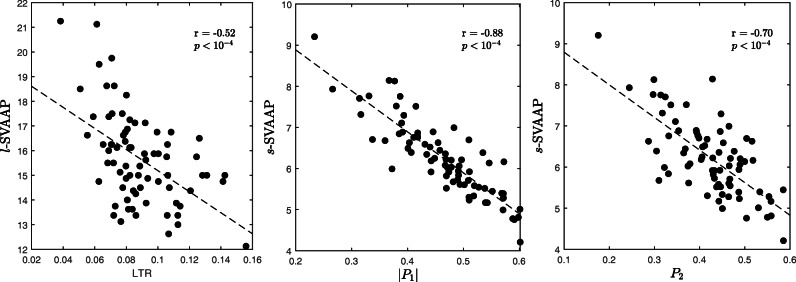
Scatter plots of *l*-SVAAP vs. LTR (left), *s*-SVAAP vs. |*P*_1_| (middle), and *s*-SVAAP vs. *P*_2_ (right). The corresponding lines of best fit and the Pearson correlation coefficients *r* are also shown

*l*-SVAAP and LTR showed a moderate negative correlation of − 0.52 (*p* < 10^−4^; Fig. 5 left). This result suggests that the more organized the overall AA is (and the less complex the AF substrate) the more uniform in time and space its propagation patterns are (fewer propagation paths), thus reaching a higher average long-term spatial correlation. Correlation between *s*-SVAAP and |*P*_1_|, and *s*-SVAAP and *P*_2_ showed negative correlations of − 0.88 and − 0.70, respectively (*p* < 10^−4^; Fig. 5 middle and right). This result suggests that on the short-term (half or full AF cycle), the more organized the AA is, the more stable its propagation patterns are. This is in agreement with the fact that a less progressed AF, which reflects in a less complex AF substrate (lower degree of electro-structural remodelling), manifests in more organized and stationary AA signals [13]. These results show the ability of *r*(*p*)^(*m*)^ curves and its characteristic indices to capture different types of information about AA dynamics during AF. Finally, when compared with each other, *s*-SVAAP was significantly lower than *l*-SVAAP (6.2(1.2) vs. 15.5(2.3), *p* < 10^−4^, median(IQR); assessed by Wilcoxon signed-rank test for paired measurements). Therefore, SVAAP tends to become smaller when quantified on smaller portions of an AA signal. This means that AA propagation patterns are overall more self-similar on a short-time period than on a long-time period, confirming the hypothesis that the overall variability in the AA propagation patterns measured on noninvasive atrial signals during persistent AF is due mainly to a spatially uncoordinated propagation of several simultaneous AF waveforms (whose spatial self-similarity decreases over progressively longer time intervals), rather than to the stable propagation of a single (or few) waveform with time-varying frequency properties (time-varying speed of propagation). This does not exclude a time-varying periodicity of the AF pattern; for instance, time-varying speed due to changes in the electrophysiological properties of the atrial tissue the waveforms travel through.

### Assessing SVAAP on the model’s output

The model gave *l*-SVAAP > *s*-SVAAP in all simulations (15(0) vs. 5.7(0.9), *p* < 10^−4^; Wilcoxon signed-rank test for paired measurements). This result supports the hypothesis that the variability observed in the AA oscillatory patterns (and thus in the AF frequency) during AF is associated with global irregular AA propagation patterns, and nonstationary spatial AA dynamics (at the whole atria level). Simulations based on completely random processes in a 15-D space gave similar values for *s*-SVAAP and *l*-SVAAP (15(0) vs. 15(0)), thus showing that the size of the segment per se does not influence the value of SVAAP, and supporting the fact that the significant difference observed for the model (and the patients) did not occur by chance.

### Relation of |*P*_1_| and *P*_2_ with LTR

**Fig. 6:**
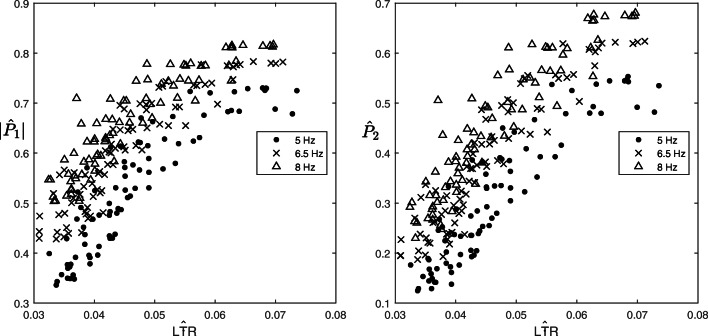
Scatter plot of $|\hat {P}_{1}|$ (left) and $\hat {P}_{2}$ (right) vs. $\hat {\text {LTR}}$, for different values of *f*_*AF*_

**Fig. 7:**
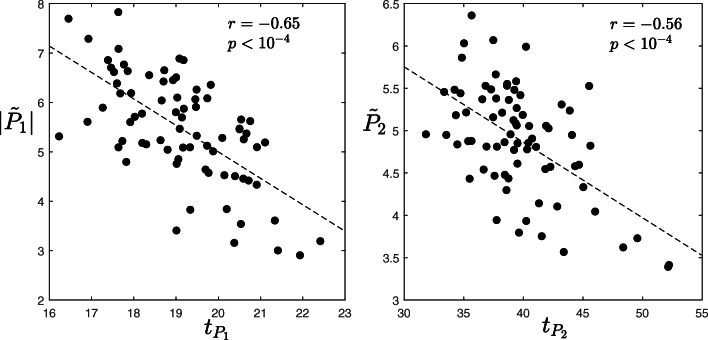
Scatter plot of $|\tilde {P}_{1}|$ vs. $t_{P_{1}}$ (left) and $\tilde {P}_{2}$ vs. $t_{P_{2}}$ (right). The corresponding lines of best fit and the Pearson correlation coefficients *r* are also shown

Figure 6 shows $|\hat {P}_{1}|$ (left) and $\hat {P}_{2}$ (right) vs. $\hat {\text {LTR}}$ for different *f*_*AF*_. $|\hat {P}_{1}|$ and $\hat {P}_{2}$ are positively correlated with $\hat {\text {LTR}}$, and this correlation is maintained for different values of *f*_*AF*_. This shows at a simulation level an influence of the AF substrate complexity (captured by LTR) on |*P*_1_| and *P*_2_. The larger the AF substrate complexity (the lower LTR) the lower the short-term recurrent behaviour of AA propagation (the lower |*P*_1_| and *P*_2_), independently of *f*_*AF*_. This is in agreement with the observation that more regular AA signals are expected with a less complex AF substrate [24]. We therefore normalized |*P*_1_| and *P*_2_ from the patients’ data accordingly, to remove this influence. Correlation between $|\tilde {P}_{1}|$ and $t_{P_{1}}$ gave a value of − 0.65 (*p* < 10^−4^; Fig. 7 left), compared with a correlation of only − 0.33 (*p* < 0.01) when |*P*_1_| is not normalized. Correlation between $\tilde {P}_{2}$ and $t_{P_{2}}$ gave a value of − 0.56 (*p* < 10^−4^; Fig. 7 right), compared with a correlation of only − 0.16 (*p* = 0.16) when *P*_2_ is not normalized. Additionally, correlation between $t_{P_{1}}$ and AF dominant frequency was − 0.86 (*p* < 10^−4^), and correlation between $t_{P_{2}}$ and AF dominant frequency was − 0.94 (*p* < 10^−4^) confirming that $t_{P_{1}}$ and $t_{P_{2}}$ are correlated to the half and full AF period, respectively, of an AA propagation waveform. Overall, this suggests that the faster the AA is propagating (the lower $t_{P_{1}}$ and $t_{P_{2}}$), the more stable its propagation paths are on the short-term (the larger the short-term spatial correlation, captured by $\tilde {P}_{1}$ and $\tilde {P}_{2}$), regardless the level of AF substrate complexity, as we have removed its influence. We can only speculate that this could be related to a cardiac tissue locally characterized by more homogeneous electrophysiological properties, which would allow for a faster and more stable propagation.

### Relation of $|\tilde {P}_{1}|$ and $\tilde {P}_{2}$ with AF recurrence after electrical cardioversion

Both $|\tilde {P}_{1}|$ and $\tilde {P}_{2}$ were significantly higher in patients not showing AF recurrence after cardioversion (5.7(1.4) vs. 5.1(1.2), *p* < 0.03, and 5.2(0.9) vs. 4.8(0.6), *p* < 0.003; Wilcoxon rank-sum test for unpaired data), suggesting a less complex AF substrate in these patients. The univariate logistic regression model generated for each index showed that both $|\tilde {P}_{1}|$ and $\tilde {P}_{2}$ are significantly associated with AF recurrence after electrical cardioversion (*p* = 0.03 and *p* < 0.01, respectively). The univariate logistic regression models for the state-of-the-art parameters showed that, for the data set used in this study, no parameter was significantly associated with AF recurrence after electrical cardioversion, as reported in Table 1. The reason for the large difference in the order of magnitude of the two indices f-wave amplitude and f-wave power is due to the different pre-processing used to compute those, as described in the original studies [2, 17].

**Table 1:** State-of-the-art parameters for the AF recurrent and non recurrent patients

Parameter	AF recurr	Non-AF recurr	*p* value
Spatial complexity (average count)	12.58 (5.17)	11.83 (5.83)	0.2209
Variability of spatial complexity (SD of count)	1.22 (1.12)	1.03 (0.85)	0.9916
Spatio-temporal stationarity (n.u.)	0.79 (0.20)	0.80 (0.18)	0.4240
Dominant frequency (Hz)	6.44 (0.79)	6.54 (0.85)	0.7784
Spectral concentration (n.u.)	0.53 (0.1)	0.49 (0.08)	0.1081
Spectral variability (n.u.)	0.42 (0.17)	0.42 (0.13)	0.4651
Multi-variate organization index (n.u.)	0.24 (0.02)	0.25 (0.03)	0.1398
Multi-variate spectral entropy (n.u.)	9.38 (0.08)	9.38 (0.09)	0.0940
Sample entropy (n.u.)	0.50 (0.02)	0.49 (0.02)	0.1976
f-wave amplitude (a.u.)	0.02 (0.01)	0.02 (0.01)	0.9732
f-wave power (a.u.)	251.06 (42.70)	244.86 (56.50)	0.4469
Harmonic energy (n.u.)	0.20 (0.08)	0.17 (0.09)	0.1446

Results are shown as median(IQR), and the *p* values from the corresponding univariate logistic regression models are also reported

The multi-variable logistic regression model showed that, when all state-of-the-art parameters are accounted for, only $\tilde {P}_{2}$ is still significantly associated with AF recurrence 4 to 6 weeks after electrical cardioversion (*p* < 0.03). This may be due in part to the fact that $|\tilde {P}_{1}|$ and $\tilde {P}_{2}$ are significantly correlated (Pearson correlation coefficient 0.83, *p* < 0.001). These results suggests that $\tilde {P}_{2}$ (and partially $|\tilde {P}_{1}|$) may provide a more sensitive measure of AF substrate complexity than state-of-the-art parameters. The lower performance of the latter may be due to the fact that all state-of-the-art parameters used in this study mainly capture the long-term recurrent behaviour in AF dynamics, unlike $|\tilde {P}_{1}|$ and $\tilde {P}_{2}$ which carry information on short-term recurrence, thus suggesting that the proposed indices may be more suitable to distinguish among patients characterized by a very similar AF substrate complexity. Finally, logistic regression on the 12-lead ECG recordings showed that only $\tilde {P}_{2}$ was close to significance (*p* = 0.049), compared with $|\tilde {P}_{1}|$ (*p* = 0.66).

## Discussion

In this study, we presented a novel framework for the noninvasive analysis of AA dynamics during persistent AF, which focuses on the recurrent behaviour of these dynamics, as observed on body surface recordings. The main motivation for this framework was the need for a methodology which could better capture the continuous spectrum of AF progression, its spatio-temporal heterogeneity and complexity, and the subtle differences in very similar AF substrates. Our findings suggest that the proposed framework allows for a detailed analysis of AA propagation dynamics during AF and for a physiological interpretation of their recurrent behaviour, and may be suitable to characterize patients with a very similar AF substrate complexity. The phenomenological model introduced to help validating this framework is simple. At the same time, it was sufficient to offer a global description of our analyses and fundamental in revealing an influence (at a simulation level) of the AF substrate complexity on the short-term recurrent behaviour of AA propagation, as observed on the body surface. This allowed us understand how to remove this influence, and achieve a better description of this short-term behaviour. Following this hint, we showed that the faster the AA is propagating, the more stable its propagation paths are in the short-term.

This is relevant since several studies over the last decade investigated how to assess the complexity of the AF substrate from the ECG, and addressed the clinical relevance of ECG-based AF substrate characterization [7, 21, 25]. Accurate noninvasive methods are needed to be able to unveil differences in patients with very similar AF substrate, likely due to structural strengthening of already existing remodelling processes [23]. Our findings suggest that the proposed framework could be used in combination with state-of-the-art ECG-based and clinical parameters to improve prediction of treatment outcome. In this respect, we showed that, for the dataset analysed in this study, state-of-the-art AF complexity parameters were not significantly associated with AF recurrence after electrical cardioversion. This suggests that these parameters are not able to properly capture the short-term behaviour of AA propagation, which is concealed by the long-term behaviour, and this may be regardless computing those parameters on smaller time windows, as almost all of them are already computed on sequential time windows of 10 s (based on the original studies). On the other hand, the proposed normalization by the long-term recurrent benaviour helps enhance the short-term information and finally obtain normalized short-term indices by which all patients have been normalized to the same level of long-term complexity, and the only remaining inter-individual variability can be associated mainly with short-term behaviours. This suggests that the proposed framework may complement the information provided by state-of-the-art measures to help their clinical applicability for AF diagnosis and treatment.

In a computational study, Manani et al. highlighted the role played by local critical patterns of uncoupling due to microstructural changes (e.g. fibrosis or gap junctional uncoupling), in anchoring microreentrant wave fronts that trigger AF [16]. This study suggests that it is the number of local critical patterns of uncoupling as opposed to global uncoupling that determines AF progression. Our hypothesis is that the long-term information about recurrence, captured by LTR, may include to some extent information on the global average uncoupling (fibrosis burden) at the level of the all atria, as reflected on noninvasive atrial recordings. Normalization of the short-term information, represented by |*P*_1_| and *P*_2_, by this global long-term information may then help highlight the contribution of local critical patterns of uncoupling, and the corresponding microreentrant wave fronts, to the normalized short-term indices $|\tilde {P}_{1}|$ and $\tilde {P}_{2}$, and better capture those microstructural changes in the myocardium. This may explain to some extent our observation that the faster the AA is propagating, the more stable its propagation paths are on the short-term (the larger the short-term spatial correlation), as fibrosis is more extensive on more progressed stages of AF (and more complex AF substrates).

In this study we focused on inference and not on prediction (or classification), and we only investigated whether the proposed indices $|\tilde {P}_{1}|$ and $\tilde {P}_{2}$ were associated with AF recurrence after electrical cardioversion in persistent AF patients. Prediction of treatment outcome and patient stratification were outside the scope of the study. A future study should look at how this novel approach could be combined with other ECG-based and clinical parameters to improve outcome prediction of electrical cardioversion and other therapies for AF. For the interested reader, the multi-variable logistic regression model assessed on the whole data set had an AUC of 84%. At the optimal operating point of the ROC curve, sensitivity was 84%, specificity 74%, and accuracy 79%.

Previous studies have used the concept of recurrence to characterize atrial wave front propagation patterns during AF and its spatio-temporal dynamics. In this respect, recurrence plots provide a way to visualize and quantify recurrent behaviour of the phase space trajectory of a dynamical system [8]. Ng et al. [20] tested the hypothesis that morphology recurrence plot analysis would identify sites of stable and repeatable electrogram morphology patterns, and found a distribution of sites with high and low repeatability of electrogram morphologies. They suggested that sites with rapid activation of highly repetitive morphology patterns may be critical to sustaining AF. Zeemering et al. [24] used recurrence plot analysis combined with principal component analysis to build a reliable tool to visualize dynamical behaviour and to assess the complexity of contact mapping patterns in AF. Both studies propose alternative ways to analyse atrial regions to detect local differences in electrogram morphology recurrence, in order to identify possible targets for ablation of AF. More recently, Almeida et al. used recurrence plots and recurrence quantification analysis to characterize the dynamics of atrial tissue activations from atrial electrograms of human persistent atrial fibrillation, and showed that this analysis allows discriminate between normal and fractionated atrial electrograms [5]. van Hunnik et al. used recurrent plots to investigate stability of conduction patterns during AF, and found that this poorly correlates with stationarity of AF properties [9]. All these studies focused on invasive recordings of AA during AF, while our study proposes the use of noninvasive recordings to assess recurrence in AA propagation during AF, and uses this information to improve quantification of AF substrate complexity. Narayan et al. [19] showed that temporal and spatial phase analysis from the ECG helps quantify progressive levels of organization in atrial and ventricular arrhythmia, and stratify intra-atrial and intra-ventricular organization during arrhythmia. Similar to their study, our study suggests that spatio-temporal regularity of AA propagation patterns can be noninvasively quantified by looking at the reproducibility of AA waveforms as recorded on the body surface, which is assessed by means of correlation-based measures (as for Eq. (1) in [19], and (1) in our study). Again, this shows the potential of surface ECG in providing subtle information about the underlying AF substrate which may be lost at a visual inspection.

The less significant association of the proposed indices with AF recurrence after electrical cardioversion obtained with the 12-lead ECG recordings extracted from the corresponding BSPM recordings suggests that a sufficiently high spatial resolution is required for the proposed framework. However, this may be improved by adding a few leads to the standard 12-lead ECG in key locations for the representation of the AA propagation patterns as reflected on the body surface. A future study should look at how much the BSPM lead system can be reduced while preserving accuracy, and how close the resulting system is to the standard 12-lead ECG. One limitation of this study is that all patients were in persistent AF. This was no arbitrary choice, as we wanted to investigate if the proposed framework could help characterize patients with very similar AF substrate. However, the suitability of this framework in both representing patients in an earlier stage of AF (as paroxysmal AF) and in distinguishing AF patients at different stages of the disease needs to be investigated in future studies. Moreover, in this study we used spatial information in BSPM recordings not in terms of locations, but with the goal of exploiting their spatial diversity, and in turn say something about the global complexity (disorganization) of the AF propagation patterns, and how disorganized over space those patterns are at the whole atria level. In this respect, potential spatial information present in BSPM recordings in terms of locations should also be investigated in a future study. Finally, all hypotheses put forward in this study on the relation between noninvasive AF recurrence and AF substrate complexity should be validated by a future study including both body surface and intracardiac recordings, and markers of structural remodelling.

## Conclusion

A novel framework for the noninvasive characterization of short-term atrial signal dynamics during persistent AF was presented. It involves the computation of an AA recurrence signal from ECG recordings which is able to separate the short- and long-term recurrent behaviour in body surface AA oscillatory patterns. The results suggest that the proposed framework, and the corresponding short-term AA recurrence measures, can be used to link the properties of body surface AA dynamics to the underlying AF substrate, and shed more light on how the progression of AF and the degree of electro-structural remodelling reflect on body surface AA signals. They also suggest that this framework can be integrated with state-of-the-art AF substrate complexity parameters to help stratify AF patients and improve selection of treatment.
